# Family of FLP Peptides in *Caenorhabditis elegans* and Related Nematodes

**DOI:** 10.3389/fendo.2014.00150

**Published:** 2014-10-14

**Authors:** Chris Li, Kyuhyung Kim

**Affiliations:** ^1^Department of Biology, City College of New York and The Graduate Center, City University of New York, New York, NY, USA; ^2^Department of Brain Science, Daegu Gyeongbuk Institute of Science and Technology (DGIST), Daegu, South Korea

**Keywords:** neuropeptides, neural circuits, behavior, nematodes, worms

## Abstract

Neuropeptides regulate all aspects of behavior in multicellular organisms. Because of their ability to act at long distances, neuropeptides can exert their effects beyond the conventional synaptic connections, thereby adding an intricate layer of complexity to the activity of neural networks. In the nematode *Caenorhabditis elegans*, a large number of neuropeptide genes that are expressed throughout the nervous system have been identified. The actions of these peptides supplement the synaptic connections of the 302 neurons, allowing for fine tuning of neural networks and increasing the ways in which behaviors can be regulated. In this review, we focus on a large family of genes encoding FMRFamide-related peptides (FaRPs). These genes, the *flp* genes, have been used as a starting point to identifying *flp* genes throughout Nematoda. Nematodes have the largest family of FaRPs described thus far. The challenges in the future are the elucidation of their functions and the identification of the receptors and signaling pathways through which they function.

## Introduction

Behavior in all animals is the output of multiple neural networks, whose individual circuits are activated, inhibited, or modulated at any one time by the combinatorial action of many neurotransmitters. In recent years, there has been a push to determine the neural connectome of organisms, including the human connectome (Human Connectome Project). The first completed neural connectome was that of the nematode *Caenorhabditis elegans* (1). In a heroic effort and with much foresight into the usefulness of the project, John White and collaborators embarked on deciphering the entire neural circuitry and mapping all chemical synapses and gap junctions between the 302 neurons of the *C. elegans* adult hermaphrodite (1); more recently, Scott Emmons and David Hall have initiated work on the *C. elegans* male neural connectome (2). The mapping of all the synaptic connections between neurons has been highly informative and helpful to researchers. However, the neural connectome, while representing a large fraction of neural network activity, underestimates the complexity of the nervous system. Layered on top of the connectome is the action of hormones and neuropeptides, whose effects can be exerted over large distances and whose actions, therefore, are not wholly represented by the neural connectome. Hence, hormones and neuropeptides add considerable complexity to neural networks to affect behavior.

In *C. elegans* and other invertebrates, neuropeptides, which are short sequences of amino acids, can act as primary transmitters as well as neuromodulators. Most neurons in *C. elegans* express at least one neuropeptide gene and in the majority of neurons, neuropeptides are co-localized with classical small molecule transmitters, such as acetylcholine, GABA, serotonin (5-HT), and dopamine (3, 4). Based on BLAST and bioinformatic screening, two major families of neuropeptides emerged in *C. elegans*: insulin-like peptides (ILPs) (5–9) and FMRFamide-related peptides (FaRPs) (10–12). In addition, *C. elegans* also has a large complement of non-insulin, non-FaRP neuropeptides (NLPs), many of which have invertebrate and vertebrate orthologs (3, 13). In this review, we will focus on the *C. elegans* FaRPs, which, by definition, all share a C-terminal Arg-Phe-NH_2_ and which are more commonly referred to as FLPs in *C. elegans*. We will describe their processing and some of their functions, and compare the diversity of FLPs in other nematodes.

## Identification of *flp* Genes

**Figure 1:**
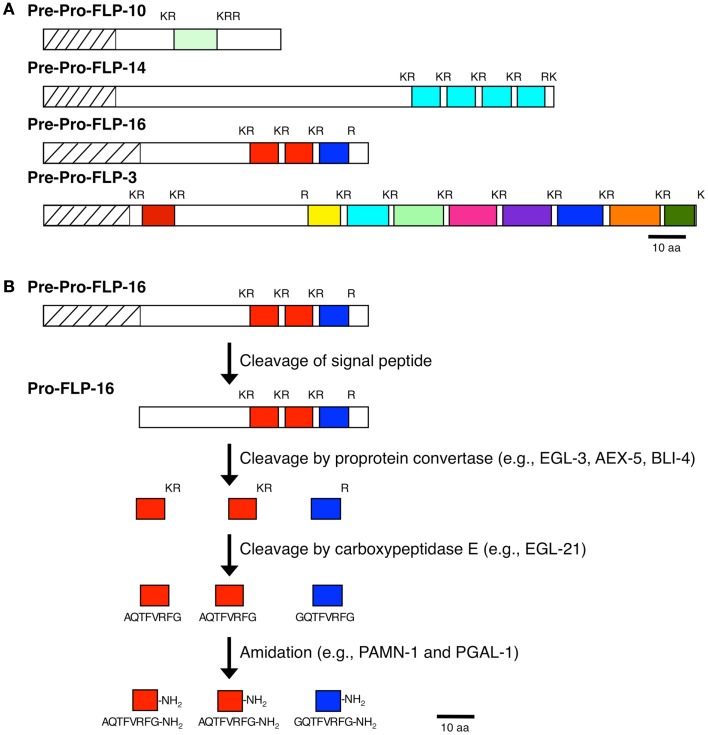
**Processing of neuropeptide precursor molecules**. **(A)** Representative FLP precursor molecules that contain one FLP peptide (FLP-10), multiple copies of the same FLP peptide (FLP-14), multiple distinct FLP peptides (FLP-3), and multiple distinct FLP peptides and multiple copies of the same FLP (FLP-16) are shown. **(B)** Proteolytic pathway of the FLP-16 precursor molecule to yield the active FLP-16 peptides.

By cDNA library screening, bioinformatic searches, and genome/transcriptome data mining for proteins with Arg-Phe-Gly sequences flanked by N- and C-terminal tri-, di-, or mono-basic residues, we and others identified 31 genes encoding FLPs in *C. elegans* (10, 12). Each *flp* gene encodes a unique set of FLPs for a total of 71 possible distinct FLPs (Table 1) (10, 12). Fourteen *flp* genes encode a single FLP, although seven of these genes encode multiple copies of the same FLP [*flp-6* (6 copies of KSAYMRF-NH_2_), *8* (3–4 copies of KNEFIRF-NH_2_), *9* (2 copies of KPSFVRF-NH_2_), *10* (1 copy of QPKARSGYIRF-NH_2_), *12* (1 copy of RNKFEFIRF-NH_2_), *14* (4 copies of KHEYLRF-NH_2_; Figure 1A), *20* (2 copies of AMMRF-NH_2_), *21* (1 copy of GLGRPLRF-NH_2_), *22* (3 copies of SPSAKWMRF-NH_2_), *24* (1 copy of VPSAGDMMVRF-NH_2_), *27* (1 copy of GLGGRMRF-NH_2_), *28* (1 copy of APNRVLMRF-NH_2_), *32* (1 copy of AMRNSLVRF-NH_2_), *33* (1 copy of APLEGFEDMSGFLRTIDGIQKPRF-NH_2_), and *34* (1 copy of ALNRDSVASLNNAERLRF-NH_2_)]. At least nine *flp* genes encode non-FLP peptides [*flp-1, 3* (Figure 1A), *7, 11, 20, 23, 26, 27*, and *34*] and six *flp* genes (*flp-1, 2, 8, 11, 22*, and *23*) are alternatively spliced (WormBase). The organization of *flp* genes ranges from the simplest at only two small exons to the largest at six exons. Similarly, the FLP protein precursors are small, ranging from 66 to 184 amino acids, sizes that include the signal peptide; the bioactive peptides vary from 5 to 24 amino acids in length (Table 1).

**Table 1:** **Predicted FLP neuropeptides in *C. elegans***.

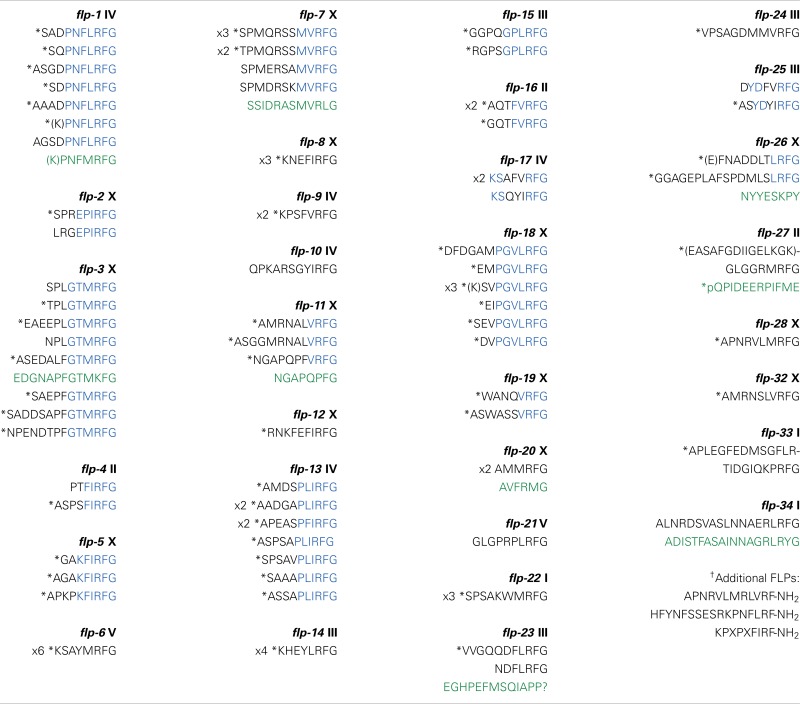

*Chromosomal location indicated after gene name. Blue indicates common amino acids among peptides encoded by the same gene; the C-terminal glycine donates an amide during amidation. Green indicates a non-FLP peptide. The number of copies of an encoded peptide is indicated*.

*Biochemically isolated. From Rosoff et al. (14), Marks et al. (15–19), Davis and Stretton (20), Li et al. (11), Nelson et al. (10), Husson et al. (21, 22), Husson and Schoofs (23), Husson et al. (24).

*^†^N. Marks, A. Maule, and A. Stretton, pers. comm*.

## Processing of Pre-Pro-Neuropeptide Precursors

As with other neuropeptides (25), the FLPs are derived from pre-propeptides (Figure 1), which are large precursor molecules that must be proteolytically cleaved and post-translationally modified to yield the active peptides (Figure 1B). The FLP precursor molecules all have a signal peptide, which is removed to yield the propeptide. The propeptides are then cleaved by proprotein convertases (PCs), serine endoproteases that preferentially recognize dibasic residues and cleave C-terminal to the basic residues (26). In *C. elegans*, PCs also cleave after tribasic and monobasic residues and very rarely, after a non-basic residue. Of the FLP precursor molecules, including different isoforms encoded by one gene, there are 184 instances of FLP sequences flanked by basic amino acids (Table 2). Seventy-one percent of the FLPs are flanked by the typical dibasic residues, of which the majority (83%) are Lys-Arg, and 29% are flanked by monobasic residues of which Arg is the most common residue (70%). Five FLP peptides are directly followed by a stop codon and one FLP peptide has an unusual non-basic flanking residue (Ala).

**Table 2:** Residues flanking FLP*.

Basic residues	No. sites
KRR	1
KR	108
RK	10
KK	9
RR	3
R	37
K	16
**Non-basic residue**
A^†^	1
Stop codon	5

*Includes all alternative transcripts.

*^†^A non-FLP peptide encoded by *flp-27* is also flanked by an A*.

*Peptides encoded by all *flp* transcripts*.

There are four genes encoding PCs in *C. elegans*: *kpc-1, egl-3/kpc-2, aex-5/kpc-3*, and *bli-4/kpc-4*; with the exception of *aex-5/kpc-3*, all are alternatively spliced to yield isoforms that differ at the C-terminus [(27, 28); WormBase], suggesting that different isoforms may have different substrate targets or preferences. Mutant alleles of all the PC genes have been isolated (27–31). By mass spectrometry (MS) analysis of neuropeptide profiles, the major PC that cleaves FLP propeptides is EGL-3/KPC-2 (22), whose mammalian ortholog is proprotein convertase 2 (PC2) (32). The number of isolated FLP peptides is significantly decreased in *egl-3/kpc-2* mutants compared to in *kpc-1, aex-5/kpc-3*, and *bli-4/kpc-4* mutants (22). However, the biochemical data also indicate that besides EGL-3/KPC-2 other PCs cleave propeptides (22). Furthermore, using an antibody that only recognizes mature FLPs, anti-FMRFamide immunoreactivity was detected in *egl-3* mutants, suggesting that other PC2s function in neuropeptide processing in *C. elegans* (33). *egl-3* is widely expressed in the nervous system and acts within post-Golgi secretory vesicles (32). Knockout of *egl-3* PC2 causes several defects, including in egg-laying (30) and body mechanosensation (32).

The minor PCs also have the capability to cleave neuropeptide precursors. *kpc-1* encodes a PC similar to mammalian furin (28) and is widely expressed in the nervous system and epithelial cells (34). *kpc-1* PC mutants show slight uncoordination and slowed growth (28) and have defects in the dendritic remodeling of the IL2 neurons during dauer formation and nictation (34). While the dendritic remodeling defects may be due to lack of cleavage of semaphorins or TGFβ substrates (34), the nictation defects may be due to lack of cleavage of neuropeptide precursors to yield active peptides that regulate nictation (35). *aex-5/kpc-3* mutants show a defect in the defecation motor pattern (31). Furthermore, knockdown of *aex-5/kpc-3* by RNA-mediated interference (RNAi) feeding gives a low rate of embryonic lethality (36), suggesting other essential substrates for AEX-5/KPC-3. The genomic organization of *aex-5/kpc-3* is slightly unusual because it is part of an operon with *unc-54*, which encodes a muscle myosin heavy chain (28); the regulation of the operon would predict that *aex-5/kpc-3* is expressed only in muscle, similar to *unc-54* myosin. However, intestinal specific expression of *aex-5/kpc-3* can rescue the defection motor program in *aex-5/kpc-3* mutants, suggesting that the intestine also expresses *aex-5/kpc-3* (37). *bli-4/kpc-4* encodes a furin-like protease that is expressed in neurons, hypodermal cells, vulval muscles, and the intestine; loss of *bli-4/kpc-4* results in late embryonic lethality (27). Although DPY-5 is a procollagen that must be cleaved by BLI-4/KPC-4 for proper adult cuticle formation (38), BLI-4/KPC-4 must have other substrates, currently unidentified, during embryogenesis. The relevance of the different PC isoforms is unknown, but may relate to substrate specificity.

The PC2s themselves are also synthesized as precursor molecules that must be proteolytically cleaved to become activated and transported (39). SBT-1 is the *C. elegans* neuroendocrine 7B2 ortholog (40) that presumably cleaves pro-EGL-3/PC2 for its activation. In high pressure liquic chromatography (HPLC)-MS isolations, 29 FLPs were isolated from extracts of *sbt-1*/7B2 mutants compared to 53 FLPs isolated from wild-type extracts, suggesting that loss of *sbt-1* interferes with neuropeptide maturation (23). *sbt-1*/7B2 mutants also showed resistance to the effects of aldicarb (40), an acetylcholinesterase inhibitor that causes paralysis in *C. elegans* (41).

After cleavage by the PCs, the C-terminal basic residues are removed by the action of carboxypeptidase E (CPE), which cleaves N-terminal to the basic residue(s). In *C. elegans*, one CPE ortholog is *egl-21*, which is expressed widely in the nervous system (33). Loss of *egl-21* CPE results in defects in egg-laying (30), locomotion, and defecation and decreased FMRFamide-like immunoreactivity and aldicarb sensitivity (33). Because residual FMRFamide-like immunoreactivity is present in *egl-21* CPE mutants (33), other carboxypeptidases must also function to process peptide intermediates. There are two other carboxypeptidases with similarity to CPE and carboxypeptidase D (33): CPD-1/F59A3.1 and CPD-2/T27A8.1^1^. Knockdown of *cpd-2*/T27A8.1 had no phenotype, whereas knockdown of *cpd-1* by RNAi showed variable uncoordination and larval lethality (42). Elucidation of *cpd-1* and *cpd-2* function in neuropeptide processing awaits future mutant analysis.

To prolong their effects, many neuropeptides are post-translationally modified to prevent their immediate degradation. In the case of the FLPs, the peptides are amidated and this amidation is required for the bioactivity of the peptide (43). The amide at the C-terminus of each mature FLP is donated by the C-terminal glycine. In mammals, amidation occurs as the result of two sequential enzymatic steps from a bifunctional protein peptidylgylcine-α-amidating monooxygenase (PAM): the peptide is subjected to the action of a peptidylgylcine-α-hydroxylating monooxygenase (PHM), followed by the action of a peptidyl- α-hydroxyglycine α-amidating lyase (PAL) (44, 45). By contrast, *C. elegans* has two independent enzymes: PAMN-1*/*T19B4.1 and PGAL-1/F21F3.1 have similarity to PHM and PAL, respectively, and could function in neuropeptide amidation [(46); WormBase].

After release, inactivation of neuropeptide signaling is regulated by their proteolytic cleavage by the class of neprilysin (NEP) zinc metallopeptidases (47). In *C. elegans*, NEP activity cleaves the amino group of hydrophobic amino acids; for instance, FLP-1 (SDPNFLRF-NH_2_) is cleaved at multiple sites (e.g., NF, FL, and RF), each including the Phe residue (48). At least 20 NEP-encoding genes are present in *C. elegans*; 10 of these are known to be expressed (48–50). *nep-1* is expressed in pharyngeal cells and a single head neuron (51), suggesting that NEPs are active at different synapses. A *nep-1* knockout showed an uncoordinated phenotype; in addition, in isolated pharyngeal preparations, *nep-1* mutants showed decreased basal activity (51). Which, if any, of these NEP genes are relevant to peptide processing is still unknown.

## Packaging and Release of FLPs

The processing of neuropeptide precursor molecules into bioactive peptides starts in the endoplasmic reticulum (ER), when the signal peptide is first removed. Subsequent cleavages of the propeptide and post-translational modifications of the cleaved peptides occur in the ER and Golgi network or as the vesicle, which in the case of neuropeptides is large, dense core vesicles (DCVs), is transported to the nerve terminal. In *C. elegans* transport of DCVs to the nerve terminals is dependent on UNC-104/KIF1A/kinesin-3 (33, 52, 53) and UNC-116/kinesin-1 (53).

Small molecule transmitters and neuropeptides are packaged into distinct vesicles, small, clear synaptic vesicles (SVs) versus large, DCVs, respectively. Calcium triggers the release of their contents with a latency of 4.5 ms after the peak of an action potential for SVs or 16.7 ms for DCVs, which show better release after repeated or prolonged stimulation (54). In addition, the contents of the vesicles are significantly different; one SV releases about 4,700 transmitter molecules with a time constant of decay of transmitter discharge of 260 μs, while one DCV releases about 18,000 molecules with a time constant of 1.6 ms (54). DCVs are marked by the presence of IDA-1/IA-2/phogrin, which is a receptor protein tyrosine phosphatase found specifically on DCVs and which regulates levels of phosphatidylinositol phospholipids (PIPs) (55, 56). Regulated release of either type of vesicle is a multi-step process: vesicle docking, priming, fusion, and release. DCVs are primarily found in *C. elegans* axons and not in cell somas; activity of the UNC-43 CaMKII regulates DCV exocytosis from the axon rather than the cell soma (57). The core SNARE complex, which includes vesicular SNB-1/synaptobrevin-1 (v-SNARE) and plasma membrane proteins RIC-4/SNAP-25 and UNC-64/syntaxin (t-SNAREs), is essential for this process and interacts with a number of other proteins, some specific to DCVs, to facilitate vesicular release (58). Presumably, v-SNARE SNT-1/synaptotagmin (59) has a similar function with DCVs and acts as a calcium sensor. Both types of vesicles also require the cytoplasmic protein UNC-18 for vesicular release; UNC-18/Munc-18 has multiple functions: (1) it helps in the efficient trafficking from the ER and anterograde transport of UNC-64/syntaxin (60); (2) it binds UNC-64/syntaxin and mediates the conformational switch of UNC-64/syntaxin from a closed to open state to allow SNARE complex assembly, thereby priming the vesicle (61–63); and (3) it allows vesicle and plasma membrane fusion (62). Unlike SVs that are docked and released from active zones, DCVs are released at sites distinct from active zones (64). These DCV docking sites may be determined by the cytoplasmic protein RIC-7, which is a nematode-specific protein with no known homologies (65). UNC-31/CAPS, a calcium-dependent activator protein that contains a DCV-binding domain and pleckstrin-homology domain, serves as a bridge between DCVs and the plasma membrane (66, 67) to promote DCV docking and release (64, 68, 69). UNC-13, which promotes SNARE complex assembly (69, 70), and PKC-1 both contain C1 domains that bind phorbol esters to regulate vesicular release, although PKC-1 is only involved in DCV release (69). UNC-13/Munc13 stabilizes the open conformation of syntaxin to promote vesicle priming and this activity is antagonized by TOM-1/tomosyn, which associates with UNC-64/syntaxin and RIC-4/SNAP-25 to form an inhibitory SNARE complex (71) and prevent DCV release (68). Unlike small molecule transmitters, neuropeptides are not recycled and excess neuropeptides are cleared from the synaptic regions by degradation (see above); new neuropropeptides must be synthesized *de novo* in the cell body, processed and packaged into DCVs, and transported to the nerve terminals.

## Isolation of FLPs and Expression Patterns

Among the 31 *flp* genes, there are 71 predicted FLPs. The initial isolation of FLPs was through laborious extracts from grams worth of animals (14–19, 21). Fortunately, the advent of new technologies with high pressure liquid chromatography in tandem with MALDI-TOF mass spectrometry have greatly decreased the amount of starting materials necessary for peptidomic studies and increased the resolution of peptide identities. Hence, microgram amounts of animals are now sufficient to isolate even low abundance peptides. The group of Liliane Schoofs has taken on the challenge to decipher the FLP peptidome in *C. elegans* and other nematodes (22, 24). Fifty-four of the 71 predicted FLPs representing 25 of the 31 *flp* genes have now been isolated (Table 1) (22, 24). However, there are also three FLPs, APNRVLMRLVRF-NH_2_, HFYNFSSESRKPNFLRF-NH2, and KPXPXFIRF-NH_2_, that have been biochemically isolated, but the corresponding gene has not been identified (N. Marks, A. Maule, and A.O. Stretton, pers. comm.). These results suggest that most, if not all, predicted FLPs are produced and additional *flp* genes have yet to be identified. Given that many of the peptides encoded by one gene are highly similar, we predict that these peptides have slightly different binding affinities to receptors to allow fine tweaking of a circuit. For instance, *flp-18* encodes six distinct FLPs that share a common C-terminal PGVLRF-NH_2_; NMR analysis of two of the peptides DGDGAMPGVLRF-NH_2_ and EMPGVLRF-NH_2_ revealed that the N-terminal aspartates of DGDGAMPGVLRF-NH_2_ form long-range electrostatic interactions with the C-terminal arginines, thereby forming a loop that decreases peptide binding to one of its receptors, NPR-1 (72). Similarly, other N-terminal extensions can form secondary structures that decrease the affinity of the peptide to its receptors, thereby modulating the biological effects of the peptides.

cDNAs from all *flp* genes have been isolated, indicating that all *flp* genes are expressed [(4, 10, 73, 74); WormBase]. Because FLPs are relatively small, ranging from 5 to 24 amino acids, and all share a C-terminal Arg-Phe-NH_2_ motif, making antibodies against specific FLPs have been difficult. A general anti-FaRP antiserum that recognizes FaRPs encoded by multiple *flp* genes showed that roughly 10% of the neurons were immunoreactive (75). This figure, however, appears to grossly underestimate the widespread expression of the *flp* genes. Using transcriptional reporters, over 50% of the nervous system was found to express one or more *flp* genes (4, 76–78). Transcriptional reporters, however, have many inherent caveats, such as incorrect/incomplete promoter regions used or intronic sequences with regulatory elements missing; in addition, researchers injecting similar constructs have sometimes reported different expression patterns (4, 77). Using a monoclonal antibody specific for FLP-8 (79, 80), we showed that the immunoreactivity pattern matched that of the reporter expression pattern, suggesting that transcriptional reporters can reflect protein expression patterns. Although each *flp* gene is expressed in a distinct set of neurons, there is considerable overlap in the expression pattern of the different *flp* genes and a single neuron can show a wide diversity of *flp* expression. For instance, the chemosensory neuron ASE and oxygen-sensor URX express five and four *flp* genes, respectively (4). In addition, most *flp* genes are expressed in neurons that also express a small molecule transmitter. Some *flp* genes are also expressed in non-neuronal cells, such as the intestine or gonad (4, 77). The complex *flp* expression pattern allows exceptionally intricate modulation of neural networks to generate behavior.

## Function of FLPs in *C. elegans*

FLP neuropeptides have been shown to function in various behaviors in *C. elegans* (Table 3). A common functional modality among FLPs is that these neuropeptides appear to inhibit circuit activity in most, but not all, behaviors tested. Pharmacological application of FLPs, however, shows that FLPs also has excitatory effects (Table 3).

**Table 3:** **Function of FLP Neuropeptides in *C. elegans***.

***flp* gene**	**Phenotypes in mutants or by RNAi**	**Phenotypes due to overexpression**	**Pharmacology of FLPs on pharynx**
*flp-1*	Loopy waveform (1); suppressed paralysis due to increased levels of dopamine (2); enhanced convulsive locomotion and aldicarb hypersensitivity in synergy with *flp-18* (3); decreased entry into the active phase of egg-laying (4)	Flattened waveform (1)	Decreased pharyngeal activity at 1 μM (5, 6)
*flp-2*			Increased pharyngeal activity at 1 μM (6)
*flp-3*			Decreased pharyngeal activity at 1 μM (5, 6)
*flp-4*			Increased pharyngeal activity at 1 μM (6)
*flp-5*			Increased pharyngeal activity at 1 μM (5, 6)
*flp-6*			Increased pharyngeal activity at 1 μM (5, 6)
*flp-8*	Increased repetitive turning during male mating (7)		Increased pharyngeal activity at 100 nM (5, 6)
*flp-9*			Decreased pharyngeal activity at 1 μM (5, 6)
*flp-10*	Increased repetitive turning during male mating (7)	Inhibits egg-laying (8)	
*flp-11*			Decreased pharyngeal activity at 100 nM (5, 6)
*flp-12*	Increased repetitive turning during male mating (7)		
*flp-13*			Decreased pharyngeal activity at 100 nM (4, 5)
*flp-14*			Increased pharyngeal activity at 1 μM (5, 6)
*flp-15*			Decreased pharyngeal activity at 1 μM (5, 6)
*flp-16*			Decreased pharyngeal activity at 1 μM (5, 6)
*flp-17*		Inhibits egg-laying (8)	Increased pharyngeal activity at 100 nM (6)
*flp-18*	Decreased odor response, increased reversals after starvation, enhanced dauer formation in *daf-7* TGFβ mutants, reduced oxygen consumption, increased intestinal fat storage (9); enhanced locomotory activity of *npr-1(g320)* mutants during lethargus (10)		
*flp-19*			Decreased pharyngeal activity at 1 μM (4, 5)
*flp-20*	Loss of massed training-induced memory for tap habituation and decreased number of synaptic vesicles (11); increased repetitive turning during male mating (7)		
*flp-21*	Blocked hypoxia-induced 5-HT stress signals from the pharynx to head neurons (12); enhanced locomotory activity of *npr-1(g320)* mutants during lethargus (10); increased thermal thresholds for heat avoidance (13); displayed aggregation behavior (14)		Decreased pharyngeal activity at 1 μM (5, 6)
*flp-22*			Increased pharyngeal activity at 1 μM (6)

*5-HT = serotonin; 1, Nelson et al. (76); 2, Wani et al. (81); 3, Stawicki et al. (82); 4, Waggoner et al. (83); 5, Rogers et al. (84); 6, Papaioannou et al. (85); 7, Liu et al. (86); 8, Ringstad and Horvitz (77); 9, Cohen et al. (87); 10, Choi et al. (88); 11, Li et al. (89); 12, Pocock and Hobert (90); 13, Glauser et al. (91); 14, Rogers et al. (78)*.

### Locomotion

Wild-type animals move in a sinusoidal waveform on a solid surface and initiate swimming in liquid. *flp-1* mutants displayed an exaggerated waveform on a solid surface; conversely, overexpression of *flp-1* caused a flattening of the waveform and extreme sluggishness, suggesting that *flp-1* modulates the locomotory circuits (76). *dat-1* encodes a dopamine transporter and mutations in *dat-1* caused paralysis due to increased synaptic dopamine concentration (92). Chase and coworkers showed that a *flp-1* mutation fully suppressed this locomotion defect of *dat-1* mutants (81). Although the exact mechanism has not been elucidated, these results further indicate a role of *flp-1* in locomotory circuits (76).

ACR-2 is a nicotinic acetylcholine receptor subunit expressed in cholinergic motoneurons (93). *acr-2* gain-of-function (*gf)* mutants exhibited excitation–inhibition imbalance due to an increase in cholinergic excitation and a decrease in GABAergic inhibition in the locomotory circuit, resulting in spontaneous convulsive behavior and increased aldicarb sensitivity (93). Loss of *egl-3* PC2 decreased GABAergic inhibition and enhanced the convulsion phenotype of *acr-2*(*gf)*, suggesting that neuropeptides were involved (82). After testing multiple neuropeptide mutants, *flp-1*; *flp-18* double mutants but not single mutants, were identified as enhancing convulsive locomotion and aldicarb hypersensitivity of *acr-2*(*gf)* by further reducing GABAergic inhibition (82). In addition, *acr-2*(*gf*) mutants showed increased expression of *flp-18* in the ventral cord cholinergic B-type motoneurons and overexpression of *flp-18* decreased convulsion phenotype of *acr-2*(*gf)* (82). NPR-1 and NPR-5 G protein-coupled receptors (GPCRs) appeared to mediate the action of FLP-18 in *acr-2* (*gf*) mutants (82). These results indicate that *flp-1* and *flp-18* play important roles in suppressing overexcitation of the locomotory circuit.

### Egg-laying

*C. elegans* egg-laying behavior is regulated by the actions of both small molecule neurotransmitters, such as acetylcholine and 5-HT, and neuropeptides (94). The HSN and VC4/5 serotoninergic neurons control the temporal switch between active/inactive states of egg-laying; during the active phase, release of acetylcholine triggers the egg-laying event (95). *flp-1* functions in parallel with serotonin to promote entry into the active phase of egg-laying (83). A different peptidergic circuit regulating egg-laying behavior was revealed by analysis of *egl-6* mutants, which were isolated on the basis of their egg-laying defects (30). EGL-6 is a GPCR; a gain-of-function mutation or overexpression of *egl-6* exclusively in the HSN motoneurons inhibited egg-laying, while *egl-6* deletion mutants did not exhibit egg-laying defects (77). However, *egl-6* deletion mutants fully suppressed egg-laying defects caused by overexpression of *flp-10* or *flp-17* (77). Surprisingly, the effects of the FLP-10 peptide were conferred by expression in non-neuronal cells, including vulval cells and spermatheca, as well as the CO_2_-sensing BAG neurons, whereas *flp-17* appeared to function primarily in the BAG neurons (77). The FLP-10/FLP-17 peptidergic circuit acted redundantly with a cholinergic circuit distinct from HSN/VCs to inhibit egg-laying (77). Using the *Xenopus* oocyte heterologous system, FLP-10 and FLP-17 were verified as cognate ligands for EGL-6 GPCR (77).

### Metabolism

de Bono and coworkers found that *flp-18* deletion mutants showed a multitude of defects, including decreased odor response, increased reversals and turns after 1 h of starvation, enhanced dauer formation in *daf-7* TGFβ mutant background, reduced oxygen consumption, and increased intestinal fat storage (87). These *flp-18* phenotypes were rescued by expression of *flp-18* in the AIY interneurons (or additionally RIG interneurons for the fat phenotype), which are major synaptic targets of multiple chemosensory neurons, indicating that the AIY neurons may integrate and process food information to determine release of FLP-18 (87). FLP-18 activated two GPCRs, NPR-4 and NPR-5, in *Xenopus* oocytes; furthermore, activation of NPR-4 in the intestine and NPR-5 in a set of chemosensory neurons mediated fat storage, activation of NPR-4 in the AVA and RIV interneurons mediated reversals and turns, and activation of NPR-5 in the ASJ chemosensory neurons mediated dauer formation (87). Fat storage was also increased in two other NPY-like receptor mutants, *npr-1* or *npr-7*, suggesting additional roles of non-FLP-18 neuropeptides in fat metabolism (87).

### Stress responses

Pocock and Hobert noticed that hypoxic stress increased levels of serotonin (5-HT) in a subset of gustatory neurons (ASG and ADF) of *C. elegans* via direct regulation by the hypoxia-inducible transcription factor HIF-1 and enhanced response to NaCl (90). Serotonin released from the ASG and ADF neurons activated the M4 pharyngeal motoneuron via its SER-7 5-HT receptor; activation of M4 resulted in the release of the FLP-21 peptide to relay these hypoxia-induced 5-HT signals from the pharynx to NPR-1-expressing neurons in the head (AQR, PQR, and URX), which may act upstream of the gustatory circuit (90). Thus, FLP-21 and its receptor NPR-1 play roles in transmitting stress signals.

### Aggregation

The wild-type laboratory strain N2 var. Bristol displays solitary feeding behavior (96). However, under stressful conditions, such as high population density, low food, or low O_2_ concentration, animals will aggregate (96–98). In addition, a mutation in the Bristol *npr-1* GPCR gene that corresponded to an NPR-1 isoform found in many naturally isolated wild strains caused aggregation even under non-stressful conditions, indicating that low activity of NPR-1 GPCR may represent stress-related behavioral states (96, 99, 100); expression of *npr-1* in the AQR, PQR, and oxygen-sensor URX neurons regulates aggregation behavior (101). Animals lacking FLP-21, a cognate ligand of NPR-1 GPCR, also displayed aggregation behavior, providing genetic evidence for interactions between FLP-21 and NPR-1 (78, 102). Recently, Bargmann, Sengupta, and coworkers found a “hub-and-spoke” circuit motif that generates behavioral differences depending upon NPR-1 activity (103, 104). In this circuit, the RMG command interneuron/motoneuron, which also expresses *npr-1* (101), serves as the hub of seven spoke neurons, including two pheromone-sensing neurons ASK and ADL and an oxygen-sensing URX, which are connected to the RMG hub via gap junctions; high RMG activity is required for aggregation behavior (103, 104). The ASK neuron is involved in male attraction and hermaphrodite repulsion at low and high concentrations of pheromone, respectively (105). High NPR-1 activity in solitary N2 hermaphrodites resulted in decreased RMG activity, leading to an avoidance response due to the enhanced ADL (repulsion) and reduced ASK (attraction) pheromone response (103, 104). Conversely, low or absent NPR-1 activity resulted in increased RMG activity, leading to an increased ASK–RMG mediated attraction and decreased ADL mediated repulsion, resulting in hermaphrodites being neutral to pheromone (103, 104). Thus, NPR-1 signaling in RMG regulates its activity level to integrate environmental signals, such as pheromones and oxygen.

### Sleep-like behavior

During each of the four larval molts, *C. elegans* exhibits a sleep-like behavioral state during a period called lethargus, during which wild-type animals show reduced sensory and motor activity and feeding behaviors (106). By contrast, *npr-1* GPCR mutants exhibited increased locomotive activity compared to that of quiescent wild-type animals; this activity was further enhanced in *flp-18* or *flp-21* single or double mutants (88). FLP-18/21 activation of NPR-1 signaling in the RMG hub-and-spoke interneuron was found to decrease sensory activity of the ASK sensory neurons, resulting in decreased PDF-1 secretion from the ASK neurons (88). Decreased PDF-1 secretion led to decreased activation of the PDF-1 receptor in mechanosensory neurons and muscles, resulting in decreased arousal (88). Thus, *flp-18* and *flp-21* have roles in decreasing neuronal and circuit activities underlying lethargus.

### Learning and memory

*C. elegans* have been shown to habituate to mechanical stimuli generated by tapping the worm plate; repeated tap stimulation caused decremented responses to taps (107). Massed training-induced memory for tap habituation lasted at least 12 h (89). This 12-h memory retention of tap habituation training was abolished in *flp-20* mutants and the increased number of SVs in mechanosensory neurons after massed training was also reduced in *flp-20* mutants, indicating that FLP-20 is required for the 12-h memory retention (89). Since *flp-20* was expressed in and acted in the mechanosensory neurons, FLP-20 may regulate the number of DCVs in mechanosensory neurons via a feedback circuit (89).

### Heat avoidance

Goodman and coworkers developed new assays to identify genes involved in heat avoidance (91). Mutations in either *flp-21* or *npr-1* were found to increase thermal thresholds of heat avoidance. These effects may be due to FLP-21 activation of the RMG interneurons to decrease thermal thresholds (91).

### Male mating

During mating, males undergo a stereotypic series of movements that includes hermaphrodite contact, backing, turning, locating the vulva, spicule insertion, and sperm transfer (108, 109). The male tail contains many specialized ray neurons that function in concert with core neurons for efficient male mating. In particular, glutamatergic mechanosensory neurons are required independently of ray neurons for turning behavior (86). In addition, loss of *flp-8, 10, 12*, and *20*, all of which are expressed in all or subsets of the mechanosensory neurons (4), increased repetitive turning behavior, suggesting that these FLPs are necessary for the accurate timing of turning (86).

### Pharyngeal pharmacology

Holden-Dye’s group has made excellent use of a dissected pharyngeal preparation that contains the pharynx and nerve ring; the pharyngeal musculature has myogenic properties that can be stimulated with 500 nM serotonin (84). By recording from the pharyngeal muscle with either an intracellular electrode or a suction pipette, the group found that numerous FLP peptides increased (FLP-17 and 8 at 100 nM; FLP-2, 4, 5, 6, 14, and 22 at 1 μM) or decreased (FLP-11 and 13 at 100 nM; FLP-1, 3, 9, 15, 16, 19, and 21 at 1 μM) pharyngeal activity (84, 85). Several of the *flp* genes with bioactive peptides are expressed within pharyngeal neurons, but many of the genes encoding the bioactive peptides are expressed in neurons outside the pharyngeal system (4). Whether the peptides are acting directly on pharyngeal muscle or indirectly via other neurons remains to be determined. In addition, because some of the peptides were bioactive only at high concentrations, the physiological roles of the peptides await further confirmation.

## Identifying G Protein-Coupled Receptors through Which FLPs Signal

With one exception (110), neuropeptides signal through GPCRs. In *C. elegans*, there are an estimated 1,100 GPCRs (111) of which most have been classified as chemoreceptors and 50–125 are likely to correspond to neuropeptide GPCRs (111, 112). Knocking down activity of a subset of the identified neuropeptide receptors suggest that signaling through these receptors affect reproduction and locomotion (113).

A current initiative by several labs has been to de-orphanize the GPCRs by identifying their respective ligand(s) and determining the pathways in which they function. This task is daunting in any system, because GPCRs are promiscuous and can bind more than one ligand; similarly, neuropeptides are also promiscuous and one peptide may bind multiple GPCRs with different affinities. Whether these differing binding affinities reflect a biological function, whereby activation by a specific peptide may only occur after low or high frequency stimulation, or are an artifact of the experimental system remains to be clarified.

Several heterologous systems have been used to de-orphanize FLP receptors: expression of receptors in *Xenopus laevis* oocytes or in cell lines and use of different signaling readouts, including whole cell voltage-clamp, GTPγS binding, cAMP levels, and calcium indicators, to determine receptor activation. The EC_50_ (concentration which produces 50% maximal activation) varies substantially among the different readouts (nanomolar to micromolar), suggesting different sensitivity among the readout systems. These studies have revealed that as with vertebrate and other invertebrate neuropeptide receptors, *C. elegans* FLP receptors are generally activated by multiple FLPs, which may be encoded by the same or, more likely, distinct *flp* genes (Table 4). FRPR-18/T19F4.1, is the only receptor identified thus far that is activated by peptides encoded by only one *flp* gene, *flp-2*, with a physiological EC_50_ (114). Most receptors are activated by peptides encoded by multiple *flp* genes. For instance, NPR-3/C10C6.2 was activated at an EC_50_ in the nM range by both peptides encoded by *flp-15* (115) and the single *flp-21* peptide, which all share a C-terminal PLRFamide (116). FRPR-3/C26F1.6 was activated by one FLP-11 and one FLP-7 peptide, both of which share a C-terminal VRFamide, while other closely related FLP-7 and FLP-11 peptides did not activate the receptor (117). EGL-6/C46F4.1 was activated by two C-terminally related peptides (YIRFamide), FLP-10, and FLP-17 (77). Several receptors can be activated by a large number of FLP peptides that do not appear to share structural similarities except for the C-terminal RFamide: NPR-22/Y59H11AL.1 binds 15 peptides encoded by six *flp* genes (*flp-1, 7, 9, 13, 11*, and 22) with an EC_50_ ranging from 25 nM to 5 μM (116, 118), NPR-4/C16D6.2 binds 13 peptides encoded by seven *flp* genes (*flp-1, 3, 4, 11, 14, 15*, and *18*) with an EC_50_ ranging from 5 nM to >10 μM (116), and NPR-11/C25G6.5 binds eight FLP peptides encoded by five genes (*flp-1, 5, 14, 18*, and *21)* with an EC_50_ ranging from 1 nM to 8 μM (116). Despite being able to activate receptors at high concentrations (e.g., EC_50_ > 10 μM), such peptides may not be physiological ligands. For instance, FLP-1 activates multiple receptors, but all at an EC_50_ that is unlikely to be physiological; hence, the FLP-1 receptor has yet to be identified. One receptor, CKR-2, has been found to bind both FLP and non-FLP peptides. CKR-2 binds NLP-12 and NLP-13 at high affinity (nanomolar range), but also binds a FLP-1 peptide at low affinity (>10 μM) (119). The complexity with which the peptides binds receptors and the receptors bind peptides suggests that behaviors can be subtly refined by modulating peptide levels.

**Table 4:** **Receptors that bind FLP peptides**.

		Activates/binds receptor		
Receptor	Cosmid	EC_50_ in nM range	EC_50_ in mM range	(EC_50_ > 10 mM)
NPR-1	C39E6.6	**FLP-18** (EMPGVLRFa, DFDGAMPGVLRFa, SVPGVLRFa, EIPGVLRFa) **FLP-21** (GLGPRPLRFa)	**FLP-18** (SEVPGVLRFa, DVPGVLRFa)	
NPR-3	C10C6.2	**FLP-15** (GGPQGPLRFa, RGPSGPLRFa) **FLP-21** (GLGPRPLRFa)		
NPR-4	C16D6.2	**FLP-4** (ASPFIRFa) **FLP-18** (DVPGVLRFa, (K)SEVPGVLRFa, SVPGVLRFa, DFDGAMPGVLRFa, EIPGVLRFa)	**FLP-1** (KPNFLRFa) **FLP-3** (SPLGTMRFa, SAEPFGTMRFa) **FLP-11** (NGAPQPFVRFa) **FLP-15** (GGPQGPLRFa)	
NPR-5	Y58G8A.4	**FLP-18** (DVPGVLRFa, (K)SEVPGVLRFa, SVPGVLRFa, DFDGAMPGVLRFa, EIPGVLRFa) **FLP-21** (GLGPRPLRFa)	**FLP-1** (KPNFLRFa, SQPNFLRFa) **FLP-3** (SPLGRTMRFa, SAEPFGTMRFa, SADDSAPFGTMRFa, EDGNAPFGTMRFa)	
NPR-6	F41E7.3			**FLP-18** (DVPGVLRFa, (K)SVPGVLRFa) **FLP-21** (GLGPRPLRFa)
NPR-10	C53C7.1	**FLP-3** (SPLGTMRFa, SAFPFGTMRFa, SADDSAPFGTMRFa, ASEDALFGTMRFa, EDGNAPFGTMRFa, EAEEPLGTMRFa) **FLP-18** (SVPGVLRFa)	**FLP-18** (KSVPGVLRFa, DVPGVLRFa, SEVPGVLRFa, DFDGAMPGVLRFa, EIPGVLRFa)	
NPR-11	C25G6.5	**FLP-18** (SVPGVLRFa) **FLP-21** (GLGPRPLRFa)	**FLP-1** (KPNFLRFa) **FLP-5** (AGAKFIRFa) **FLP-14** (KHEYLRFa) **FLP-18** (KSVPGVLRFa)	
NPR-22	Y59H11AL.1		**FLP-7** (SPMERSAMVRFa)	**FLP-1** (KPNFMRYa) **FLP-7** (TPMQRSSMVRFa, SPMQRSSMVRFa, SPMDRSKMVRFa) **FLP-9** (KPSFVRFa) **FLP-11** (AMRNALVRFa, NGAPQPFVRFa) **FLP-13** (AADGAPLIRFa, ASPSAPLIRFa, SPSAVPLIRFa, ASSAPLIRFa, SAAAPLIRFa) **FLP-22** (SPSAKWMRFa)
FRPR-3	C26F1.6		**FLP-7** (TPMQRSSMVRFa) **FLP-11** (AMRNALVRFa)	**FLP-7** (SPMQRSSMVRFa, SPMERSAMVRFa)
FRPR-18	T19F4.1	**FLP-2** (SPREPIRFa, LRGEPIRFa)		**FLP-10** (QPKARSGYIRFa) **FLP-11** (AMRNAVLRFa) **FLP-14** (KHEYLRFa)
EGL-6	C46F4.1	**FLP-10** (QPKARSGYIRFa) **FLP-17** (KSQYIRFa, KSAFVRFa)		
CKR-2	Y39A3B.5	**NLP-12** (DYRPLQFa, DGYRPLQFa)		**FLP-1** (SADPNFLRFa) **NLP-13** (pQPSYDRDIMSFa) **NLP-14** (ALDGLDGSGFGFD)

*a, amidated, p, pyroglutamic acid*.

*From Ringstad and Horvitz (77); Rogers et al. (78); Cohen et al. (87); Kubiak et al. (102), Mertens et al. (114); Kubiak et al. (115); Lowery et al. (116); Mertens et al. (117); Mertens et al. (118); Janssen et al. (119); Kubiak et al. (120); Larsen et al. (121)*.

## Diversity of FLPs in Nematodes

The accessibility of *C. elegans*, ease of manipulation and maintenance in the laboratory, and wealth of previous research have made *C. elegans* a useful model in the study of FLPs in other nematodes, and, in particular, parasitic nematodes. Parasitic nematodes infect over 1 billion people worldwide (University of Cambridge) and contributes to greater than $126 billion of crop damage globally (122). The recent sequencing of multiple nematode genomes and transcriptomes has greatly enhanced our understanding of the FLP complement in different nematodes. In addition to *C. elegans*, there are now 16 nematode genomes and/or transcriptomes that have been completed, although some are not as well annotated as others; this collection includes both parasitic and non-parasitic nematodes^2^. Other nematode sequences have also been deposited in the NCBI database, allowing us to use bioinformatic techniques to scan for *flp* genes among these genomes. By comparison to the *C. elegans flp* genes, there are 30 parasitic and 6 free-living nematodes that have *flp* orthologs [(123); unpubl. obs.].

Some striking observations can be noted among this collection of sequences. First, most nematodes, including free-living and parasitic (animal and plant), have a large complement of *flp* genes (Table 5). The most frequently appearing genes (>65%) among the scanned genomes are *flp-1, 11, 14*, and *18*; other common genes (>50%) include *flp-6, 12, 13, 16, 19, 21, 22*, and *24*. There were also some genes that appear infrequently. For instance, *flp-10* has only been identified in free-living nematodes (both hermaphrodite and male/female species), whereas, *flp-31* only appears in plant parasitic nematodes (123). Among the nematodes whose genomes have been completely determined, free-living and animal parasitic nematodes express *flp-8, 15, 23, 24*, and *33*; none of the plant parasitic nematodes have been found to express these *flp* genes thus far. By contrast, we have not identified any *flp* gene that is expressed in free-living and plant parasitic nematodes, but not in animal parasitic nematodes. Some *flp* genes appear in free-living and parasitic nematodes, but are poorly represented, appearing in <25% of the genomes scanned (e.g., *flp-9, 26*, and *28*). As the genomes/transcriptomes become better annotated, these numbers may change. However, the diversity and number of FLPs suggest that FLPs are widely used as neuromodulators in many, if not all, nematodes.

**Table 5:** **FLP diversity in nematodes**.

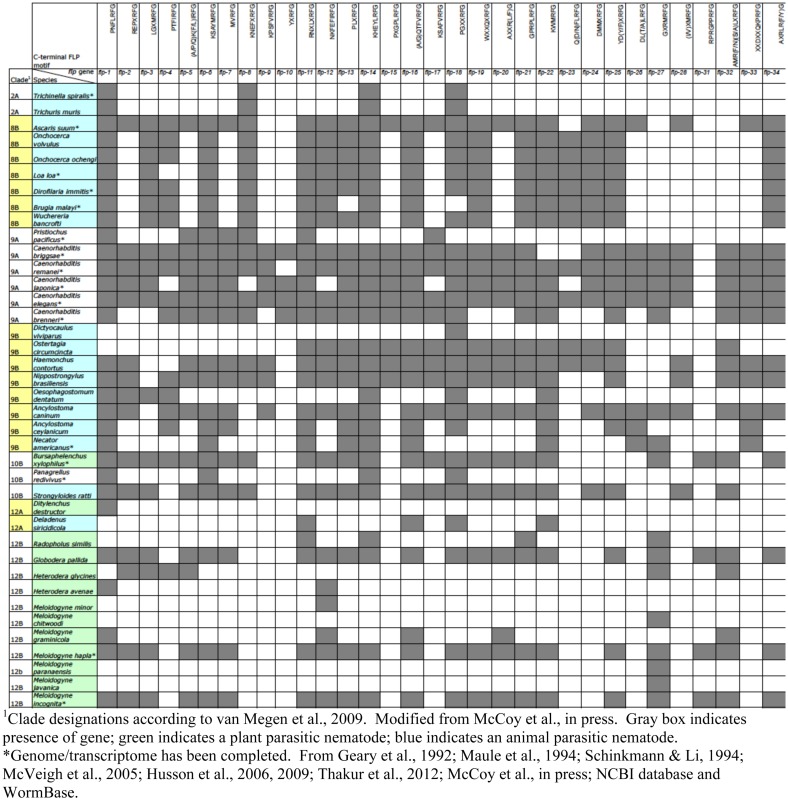

### Function of FLPs in non-*C. elegans* nematodes

Because of the difficulty of propagating some of the parasitic nematodes in the lab, the function of peptides has been difficult to query. Furthermore, many genetic tools, such as isolating mutants or generating transgenic strains, are not available. However, the larger sizes of some nematodes allow the ability to do dissections and perform electrophysiological recordings and *in situ* hybridization techniques (128–130). Hence, the *flp* expression patterns described in many pathogenic nematodes based on *in situ* data may more accurately reflect the expression patterns of the genes than in *C. elegans*, where expression patterns are generally determined by use of transcriptional reporters (12). In addition, while the decreased expression of the SID-1 receptor in neurons hampers analysis of neuropeptides in *C. elegans* (131, 132), RNAi by soaking has been effective in neurons of some pathogenic nematodes (133). However, as in *C. elegans*, there are many caveats with the use of RNAi, particularly as there are no mutants among parasitic nematodes that can be used to confirm RNAi phenotypes (134). For simplicity in the discussion below, any *C. elegans* FLP peptide that is identical and conserved through different nematode species will be referred to by the *C. elegans* name, while peptides that are specific to a certain species will be indicated as such.

### Locomotion

The neuromuscular system is highly sensitive to disruptions in FLP peptide levels. In a sand column motility assay, knockdown of *Gp*-*flp-1* and *18* and *flp-6, 12*, and *14* inhibited motility of second-stage juvenile (J2) *Globodera pallida* animals by 70–100% after 24 h of dsRNA soaking; the effects were transitory and by 6 days post-treatment, 27.1% of the animals recovered 39.4% of their normal activity (133). By contrast, when *Gp*-*flp-32* was knocked down, *G. pallida* and *Meloidogyne incognita* juveniles became more mobile, which translated to an enhanced ability to infect potato plant and other plant roots (135).

### Chemosensation

When infecting the roots of tomato plants, RNAi knockdown of *Mi-flp-18* in J2 *M. incognita* dramatically decreased chemotaxis to tomato plant roots, thereby decreasing rates of infection (136). The exudate of *Chrysanthemum coronarium*, which is often planted with tomato, contains lauric acid, which decreases *Mi-flp-18* expression, hence providing a mechanism to alleviate crop damage (136).

### Pharmacology of FLPs

Muscle preparations of some of the larger nematodes have allowed the pharmacological application of different FLPs at nanomolar to micromolar concentrations. For instance, *Ascaris suum* somatic muscle strips can be isolated, muscle tension recorded, and the effects of different FLPs examined (126). *As*-FLP-18 (0.1 μM) caused contraction and occasionally rhythmic activity; this action could be reversed by FLP-1 (SADPNFLRF-NH_2_; 0.1 μM) (137). *As*-FLP-1, FLP-1 (SADPNFLRF-NH_2_, SDPNFLRF-NH_2_; 1 nM), FLP-13 (APEASPFIRF-NH_2_;10 μM) caused relaxation of muscle strips whether innervated or denervated (16, 138). Furthermore, *As*-FLP-1 (1 nM) inhibited contractions of spontaneously active preparations (138) and hyperpolarized muscle membrane (139). Some of the FLPs had more complex actions and produced biphasic responses. FLP-8 (0.1–1 μM) and FLP-14 initially caused relaxation, followed by rhythmic contractions; FLP-14 (nanomolar) was 10–100 times more potent on the muscle strips than FLP-8 (138, 140). FLP-6 (0.1–10 μM) caused a rapid (within 30 s) increase in muscle tension in ventral muscle strips, but relaxation of dorsal muscle strips (126, 138). Intracellular recordings from *A. suum* muscle support the effects of some FLPs on muscle strips. Namely, FLP-1 (SDPNFLRF-NH_2_ and SADPNFLRF-NH_2_; EC_50_ ~300 nM) caused a slow hyperpolarization and decreased amplitude of the excitatory junction potentials (141).

The reproductive system of *A. suum* also provides a robust biological system to examine the activity of multiple FLPs. In particular, the ovijector contains circular muscle that regulates egg release and sperm influx; the muscle is innervated by a nerve plexus, which is FMRFamide-like immunoreactive (142). When the ovijector was hooked to a photo-optic transducer, the ovipositor revealed rhythmic spontaneous contractions (143). Most FLPs induced an inhibitory response, whereby there was a decrease in contraction frequency and amplitude; these peptides included FLP-3 (SPLGTMRF-NH_2_; 0.1–10 μM), 4 (ASPSFIRF-NH_2_; 0.01 μM), 7 (SPMERSAMVRF-NH_2_; 0.1 μM), 10 (QPKARSGYIRF-NH_2_; 1 μM), 11 (NGAPQPFVRF-NH_2_; 1 μM), 12 (1 μM), 13 (ASSAPLIRF-NH_2_; 1 μM), 15 (GPSGPLRF-NH_2_; 0.1 μM), FLP-16 (GQTFVRF-NH_2_; 0.1 μM), 17 (KSQYIRF-NH_2_; 0.1 μM), and 20 (AMMRF-NH_2_; 10 μM) (143). A few FLPs caused an excitatory response [FLP-2 (SPREPIRF-NH_2_; 10–100 nM), 18 (SVPGVLRF-NH_2_; 10 nM–10 μM), 19 (WANQVRF-NH_2_; 10 nM–10 μM; ASWASSVRF-NH_2_; 10 μM), and 21 (1–10 μM)] or showed a shortening of the ovipositor before an increase in contraction frequency [FLP-11 (AMRNALVRF-NH_2_; 100 nM)] (143). The activities of some peptides were only a transient contraction [FLP-2 (LRGEPIRF-NH_2_; 0.1–10 μM)] or a transient contraction followed by an extended period of inactivity [FLP-5 (AGAKFIRF-NH_2_, APKPKFIRF-NH_2_), 8, and 22 (SPSAKWMRF-NH_2_)] (143). Whether these FLP responses are physiological are unclear, particularly as some activities were only induced at high, micromolar concentrations.

To determine the effect of peptides in intact animals, peptides were directly injected into the *Ascaris* pseudocelom (injected at 100 μM; final concentration within pseudocelom estimated at 10 μM). FLP-8 and 14 inhibited locomotion, caused shallower waveforms with no propagation, and shortened body length, while injection of FLP-4 (ASPSFIRF-NH_2_), 6 (KSAYMRF-NH_2_), 7 (SPMQRSMVRF-NH_2_), 13 (APEASPIRF-NH_2_), and 16 (AQTFVRF-NH_2_) and *As*FLP-13 abolished body waves and increased body length; *As*-FLP-1 produced a more severe phenotype by causing a complete paralysis (144, 145). By contrast, injection of *As*-FLP-18 increased the number of body waves, but the body waves did not propagate and body length was decreased (145). FLP-6 and 9 caused ventral coils, while FLP-12 caused uncoordination (145).

### Physiology of FLP peptides on *A. suum* motoneurons

Several FLPs are expressed in the motoneurons of many nematodes. *A. suum* presents a relatively accessible system in which DE2 excitatory and DI inhibitory motoneurons can be exposed *in situ* and recorded while saline containing test solutions (generally 10 μM except where noted) are superfused; recorded responses can be direct actions of peptides on motoneurons or indirect effects via presynaptic neurons (128, 146). FLP-8 (1–100 nM) abolished slow oscillatory membrane potentials and reduced input resistances (147). *As*-FLP-4 and FLP-8 induced strong depolarizations in DE2 and weak depolarizations in DI motoneurons and generally decreased input resistances; FLP-16 elicited strong depolarizations in DE2 and DI (144). FLP-14 induced a stronger depolarization, but showed a transient increase in input resistance followed by a longer decreased input resistance in DE2 neurons; although FLP-14 caused a modest decrease in the input resistance of DI motoneurons, there was no membrane potential effect (144). The depolarizations elicited by the FLPs were in many cases the result of increased frequency of EPSPs. FLP-21 caused a complex response in which there was an initial hyperpolarization, followed by a sustained depolarization in DE2 neurons, but only a hyperpolarization in DI neurons; no change or a decrease was seen in the input resistance of DE2 and DI neurons, respectively. Application of different *As*-FLP-18 peptides or *As*-FLP-1 elicited weak depolarizations in DE2. FLP-6 elicited a weak hyperpolarization in DE2, but a strong depolarization, followed by miniature IPSPs in DI (144). *As*-FLP-13 elicited weak hyperpolarizations in DE2 and DI motoneurons. No effect was seen with application of *As*-FLP-28 in DE2 or DI neurons. As seen in other *Ascaris* preparations, FLPs exert a diversity of effects, but the physiological relevance has yet to be determined.

## Summary

The diversity and plethora of neuropeptides that can act over long distances allows invertebrates to greatly increase the complexity of their neural networks despite the relatively small number of neurons. A single neuron may mediate response to several sensory modalities and activate different or conserved downstream pathways to ensure correct motor outputs. Despite the relatively simple nervous system of *C. elegans*, the clever use of neuropeptides, gap junctions, hub neurons, and conventional synaptic connections allows the animal to have a wide behavioral repertoire that can be finely tuned in response to different environmental stimuli. Understanding how these simple neural networks determine behavior remains the challenge for the future.

## Conflict of Interest Statement

The authors declare that the research was conducted in the absence of any commercial or financial relationships that could be construed as a potential conflict of interest.

## Footnotes

^1^http://www.wormbase.org

^2^http://www.nematodes.org
